# Leveraging Accelerometer Data for Lameness Detection in Dairy Cows: A Longitudinal Study of Six Farms in Germany

**DOI:** 10.3390/ani13233681

**Published:** 2023-11-28

**Authors:** Anastasia I. Lavrova, Alexander Choucair, Andrea Palmini, Kathrin F. Stock, Martin Kammer, Friederike Querengässer, Marcus G. Doherr, Kerstin E. Müller, Vitaly Belik

**Affiliations:** 1 System Modeling Group, Institute of Veterinary Epidemiology and Biostatistics, School of Veterinary Medicine, Freie Universität, 14177 Berlin, Germany; 2 Ruminant and Swine Clinic, School of Veterinary Medicine, Freie Universität Berlin, 14177 Berlin, Germanykerstin-elisabeth.mueller@fu-berlin.de (K.E.M.); 3 Vereinigte Informationssysteme Tierhaltung e.V., Heinrich-Schröder-Weg 1, 27283 Verden (Aller), Germany; 4 LKV Bayern e.V., Landsberger Straße 282, 80687 München, Germany; 5 Institute of Veterinary Epidemiology and Biostatistics, School of Veterinary Medicine, Freie Universität, 14177 Berlin, Germany

**Keywords:** dairy cattle, lameness, locomotion score, machine learning

## Abstract

**Simple Summary:**

Lameness is a common problem among dairy cows, significantly impacting both their well-being and productivity. It not only hampers the mobility of cows but also exerts notable effects on behavioral aspects, like grooming and feed bunk visits. Typically, dairy cows are equipped with accelerometers primarily for estrus detection. Our goal was to formulate a model for detecting lameness using accelerometer data collected from six farms in Germany. Various statistical models were developed for lameness detection, including variables influenced by lameness, such as rumination, feeding and movement patterns, milk production, days in milk, and weight. The aim was to identify lameness in cows through a comprehensive analysis. We explored multiple models, considering different variables affected by lameness, and ultimately selected the most suitable model.

**Abstract:**

Lameness in dairy cows poses a significant challenge to improving animal well-being and optimizing economic efficiency in the dairy industry. To address this, employing automated animal surveillance for early lameness detection and prevention through activity sensors proves to be a promising strategy. In this study, we analyzed activity (accelerometer) data and additional cow-individual and farm-related data from a longitudinal study involving 4860 Holstein dairy cows on six farms in Germany during 2015–2016. We designed and investigated various statistical models and chose a logistic regression model with mixed effects capable of detecting lameness with a sensitivity of 77%. Our results demonstrate the potential of automated animal surveillance and hold the promise of significantly improving lameness detection approaches in dairy livestock.

## 1. Introduction

Lameness, characterized as impaired locomotion, is a sign associated with numerous disorders of the locomotor tract, predominantly affecting the digits or the distal part of the limb [1,2]. The evaluation of movement impairment in lame cows involves visual mobility scoring, taking into account aspects such as the back line, stride length, duration of the supportive phase, and demonstration of head bobbing during walking [3]. Generally, the state of lameness is multifactorial in origin, influenced by risk factors such as the transition cow period, nutrition, parity, and milk yield [1,2,4].

Lameness not only hampers the mobility of cows but also significantly impacts behavioral patterns, such as grooming and resting behavior. Impaired mobility leads to reduced dry matter intake and fewer visits to the feed bunk [5]. In a recent review [4], a meta-analysis considering more than 100 papers was performed in order to identify the main risk factors associated with lameness occurrence; see also [6]. The key risk factors highlighted include claw overgrowth, the first 120 days in milk (DIM), and herd size. In addition, housing conditions, including cubicle dimensions, neck curb height, walking space design, and climatic and hygienic factors, impact claw health [2,7]. Currently, visual mobility scoring stands as the standard method for detecting lame cows [8,9]. Given that lameness reflects pain, early identification and treatment of lame cows are crucial for ensuring animal welfare. A recent review [9] explored different accelerometer-based systems, identifying variables such as the number and duration of lying bouts and gait characteristics as valuable for lameness detection.

Automatically recorded behavioral parameters, such as daily bout duration, the amount of activity impulses per hour (AI/h) or per day, and daily milk yields, exhibit the potential to identify pain and discomfort, serving as predictors of an unhealthy animal [8]. In addition to lameness, other diseases, like abomasal displacement, ketosis, mastitis, and metritis, can induce changes in activity and milk yield [10,11]. Moreover, animal-related factors such as DIM and parity may contribute to variations [12,13] in activity.

The accuracy of distinguishing between lame and nonlame cows is contingent on the type of device employed, whether it be a pedometer, accelerometer, or video-based system [8,9]. Despite this variability, sensor technology designed for recording activity patterns, particularly for estrus detection, is already widely implemented on dairy farms [14,15,16]. This widespread use facilitates the acquisition of ample data for subsequent processing and the development of statistical models for lameness detection and prediction.

There are plenty of recently developed mathematical methods for data analysis in the context of lameness detection and prediction, e.g., [17,18,19]. In general, a range of statistical models, including linear and logistic regression models, have been formulated, incorporating variables associated with lameness, such as rumination, feeding and movement patterns, milk production [20], days in milk, or weight [17,21,22]. These models aim to achieve high accuracy in detecting lameness in cows. More advanced models have employed image analysis techniques, particularly wavelet analysis [18,23,24], utilizing data obtained from specialized video-based systems. In addition to the factors mentioned, three additional indicators suitable for lameness detection—back posture, milk peak flow rate, and milk peak conductivity—have been identified [24]. Wavelet analysis of cow activity and somatic cell counts in milk has been proposed for the detection of both lameness and mastitis, showing potential for individual disease detection in dairy cows, though further analysis is needed [19]. Another approach [23] involves principal component analysis of pedometer and lactation time series to detect early stages of mastitis and lameness, though this method poses challenges in results interpretation.

Thus, there are quite a lot of studies devoted to the detection of lameness based on changes in cow behavior and milk yield. However, many of these studies have been conducted on experimental farms with relatively small sample sizes, often overlooking individual cow effects on a personal level [8,9,25]. Moreover, the variation in external conditions, such as seasonal changes, is seldom taken into account in these investigations.

In this study, we investigated an original dataset collected from six farms in Germany. The primary objectives of our investigation were (1) to find out the variations in activity and productivity indicators between lame and nonlame cows; (2) to assess the significance of external factors, specifically considering seasons; and (3) to develop lameness detection models and evaluate their quality in terms of sensitivity (SN), specificity (SP), and accuracy (ACC).

## 2. Materials and Methods

### 2.1. Animal Management and Housing

The dataset comprises information gathered from six dairy farms in Germany, involving a total of 4860 cows housed in a loose-housing system. Data collection occurred between May 2015 and October 2016. To enlist participant farms, contact lists provided by the accelerometer-distributing company Lemmer-Fullwood GmbH (Lohmar, North Rhine-Westphalia, Germany) were utilized. Farms were approached and invited to voluntarily join the study. Selection criteria required lactating cows to be equipped with accelerometers (Fullexpert^®^ Differential Precision Pedometer, Lemmer-Fullwood GmbH, Lohar, North Rhine Westphalia, Germany) and stalled throughout lactation in free stall barns without access to pasture. The housing and management practices were assessed once, adhering to the Animal Needs Index 200 criteria [26].

### 2.2. Locomotion Assessment and Daily Milk Data

The locomotive behavior of the cows was evaluated using a 5-point numerical rating system (NRS) with increments of 1 [3], on a fortnightly basis whenever feasible. To conduct the assessment, cows from each pen were gathered at the end of a straight walking alley. An assistant separated each cow individually from the group, and if a cow showed reluctance to walk, gentle encouragement was provided. Observers were stationed approximately 20 m apart from the group, positioned on the right side of the walking alley in the direction of the cows’ movement. For the assessment, each approaching cow was halted and then walked by the observers. The scoring took place over distances ranging between 20 and 30 m to ensure accurate evaluation of the arch of the back, the gait, and the cow’s ability to bear weight while walking and standing, aligning with the criteria specified for correct locomotion [3].

The accelerometer, mounted on the cow’s leg, measures impulses exerted by vertical forces and its orientation along the *x*-, *y*-, and *z*-axis, allowing the classification of a cow’s activity as standing, lying, or walking [27]. The sensitivity of the device lies within a 5% deviation [28]. Throughout lactation, lactating cows were equipped with one device, at either the metatarsus or the metacarpus. In instances where a device was lost, the farmer replaced it. Data transmission occurred in the milking parlor, and data were sent to the farmer’s computer. The collected data were individually stored for each cow in the dairy management program FULLEXPERT^®^ (Lemmer-Fullwood GmbH, Lohmar, North Rhine-Westphalia, Germany). Software then processed the raw data, providing mean activity impulses per hour (AI/h) and mean lying bout duration in minutes.

Daily milk yields were automatically recorded by the milking machines (Lemmer-Fullwood GmbH, Lohmar, North Rhine-Westphalia, Germany). To ensure comparability across all farms, precise milking times and intervals were taken into account when deriving daily yields. The Federal Milk Control Councils of Berlin-Brandenburg, Saxony, and Bavaria provided basic data for each cow, including sex, breed, date of birth, and performance data, such as calving date and parity. These details were obtained through the responsible computation centers (Rinderdatenverbund (RDV), Munich, Bavaria, Germany, and IT Solutions for Animal Production (vit), Verden, Lower Saxony, Germany).

### 2.3. Data Description

The obtained data included individual-cow factors, including mean daily activity impulses per hour (AI/h), average lying bout duration (in minutes), parity, lactation stage (DIM), locomotion score [3], and daily milk yield (in kg). Visual locomotion scoring was performed 29,481 times (as described in [3]) at intervals of 2 weeks on 12 occasions within 180 days out of a total of 511 days (from 31 May 2015 to 23 October 2016). In total, locomotion scoring was performed on 60 distinct days. Cows assigned Score 1 or 2 were categorized as nonlame (0), while those assigned Score 3, 4, or 5 were considered lame (1). Despite continuous activity measurement (with occasional gaps), the final processed dataset, following the principle of parsimony, included information for the 3 consecutive days preceding the day of locomotion scoring. This information comprised average daily activity impulses, daily averaged lying bout duration, and milk yield. Expanding the time frame to 7 consecutive days preceding the day of locomotion scoring did not yield significant changes in the results (not shown here).

The observations represent repeated measures across different, nonsubsequent time periods, involving a total of 2757 units (cows) after data cleaning. On average, each cow received a lameness score 8 times (median), with a maximum of 14 times and a minimum of once. After handling missing values, a total of 20,247 measurements were utilized for model development. Furthermore, values exceeding the upper 2% quantile for activity impulses, lying bout duration, and milk yield were excluded as they were deemed infeasible (e.g., daily milk yield exceeding 80 kg).

### 2.4. Statistical Modeling

To predict lameness, we will employ a logistic-regression-based approach. In this method, we dichotomize the locomotion score, considering scores below 3 as indicative of nonlame cows and scores equal to or higher than 3 as indicative of lameness. Given that animals were frequently scored at various time points, we will incorporate random effects for cow-individual factors, selecting farms as categorical variables. The logistic regression model is formulated as follows:

(1) $$\log\left(\frac{\pi }{1-\pi }\right)=x_{i}\beta +u_{ij}+\varepsilon ,$$

where β is a vector of regression coefficients (fixed effects) and $x_{i}$ is a matrix of major independent or explanatory time-dependent variables listed in Table 1; $u_{ij}$ are (nested) random (intercept) effects of farm unit *j* and unit (cow) *i*; and ε is an error vector (unobserved), which is assumed to be uncorrelated with the random effects vector. $\pi =\mu_{y}$ is a conditional mean (i.e., the probability that the target variable $y_{ij}=1$ (lame) provided the existing $x_{i}$ values). Then, $\left(\pi /(1-\pi )\right)$ gives us the odds ratio that $y_{i}=1$ and $\log\left(\pi /(1-\pi )\right)$ is *log odds* or *logit*.

The quality of fit for the model will be estimated using the formulas for accuracy ($Acc=\left(TP+TN\right)/(TP+TN+FP+FN)=60.2\%$), specificity ($Sp=TN/(TN+TP)=82.3\%$), and sensitivity ($Sn=TP/(TP+BN)=86.4\%$). Here, TP and TN are numbers of true-positive and true-negative results, respectively, while FP and TN are numbers of false-positive and false-negative results, respectively. The criteria for an appropriate model choice include model accuracy, $R^{2}$ value, and the Akaike information criterion (AIC). The data processing involves a self-written code in Python 3.7 [29], and the statistical model is implemented in R 4.0.3 [30].

## 3. Results

### 3.1. Data Processing

**Figure 1:**
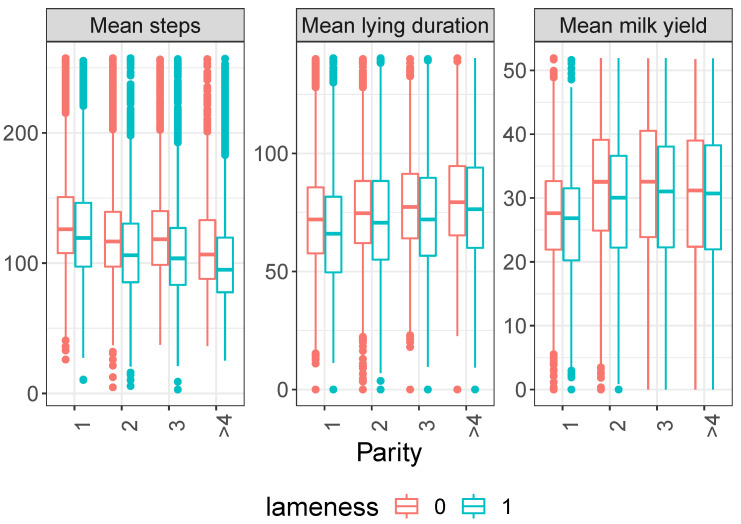
Daily average number of activity impulses per hour (**left**), average lying bout duration (min, **middle**), and daily milk yield in kg (**right**)—all three indicators averaged over 3 days before locomotion scoring for lame (locomotion score ≥3) and nonlame (locomotion score <3) cows, categorized by cow parity. Red and blue colors represent nonlame (0) and lame (1) cows, respectively.

**Figure 2:**
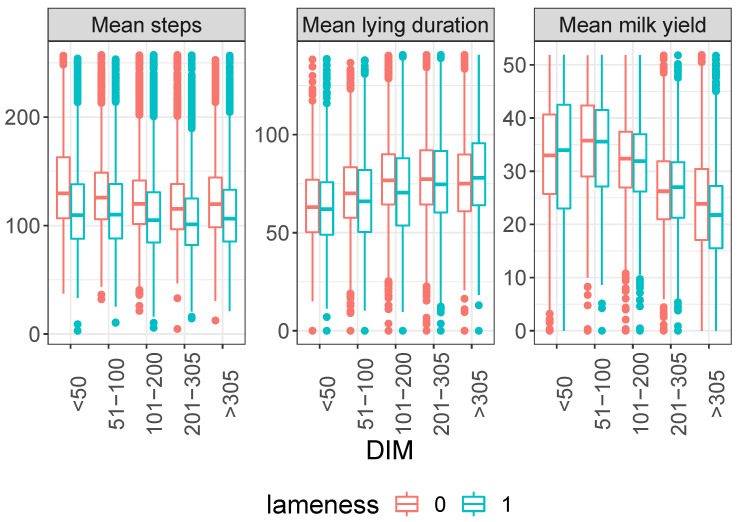
Averaged daily number of activity impulses per hour (**left**), lying bout duration (**middle**), and milk yield (**right**) over 3 days preceding locomotion scoring. Data presented for cows with different lameness statuses (lame: locomotion score ≥3; nonlame: locomotion score <3), plotted against days in milk (DIM).

In Figure 1 and Figure 2, box plots illustrate the distribution of activity (measured by daily activity impulses), duration of a single lying bout (lying duration), and milk yield based on individual cow factors, such as parity and days in milk. Notably, differences in activity and productivity indicators (mean activity impulses, mean lying bout duration, and mean milk yield) are observed at a statistical level but not for individual measurements. Additionally, the data are not normally distributed (confirmed by the Shapiro–Wilk test on some of our data), and medians for lame and nonlame cows show slight differences. However, a substantial overlap in both interquartile intervals and outliers makes it challenging to distinguish between lame and nonlame animals.

**Figure 3:**
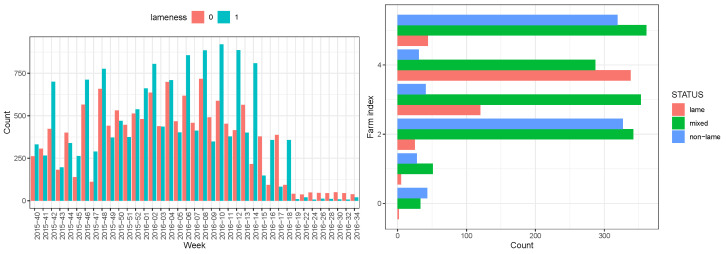
Numbers of lame and nonlame cows per observation and over time. (**left**) The cases of lameness scoring per week represented by a histogram. Nonlame (0) and lame (1) statuses are shown by color (red and blue, respectively). (**right**) Distribution of cows categorized as persistently lame, persistently nonlame, and mixed, based on locomotion scoring results from six farms. Cows with alternating lame/nonlame states were classified as mixed. The colors green, red, and blue correspond to mixed, persistently lame, and persistently nonlame cows, respectively.

The dataset indicates that lameness is a serious problem affecting 50±2.2% of animals, representing the average percentage of animals scored as lame. It is worth noting that animals could be scored multiple times on different days, as shown in Figure 3. The number of lame cows varies depending on the season and the farm. In Figure 3 (left), the counts of lame and nonlame cows from six dairy farms, determined by visual locomotion scoring [3], are presented based on the scoring week. The lower number of scorings towards the end of the study period was due to a delayed start of the observations and a postponed end of the study on a single farm. Furthermore, the duration of lameness episodes was investigated, categorizing cows into three different status groups based on repeated lameness scorings: persistently lame (score 3 and higher at all observations), persistently nonlame (scores 1 and 2 at all observations), and mixed group (scored interchangeably as lame and nonlame). A histogram depicting the distribution of these three groups across different farms is presented in Figure 3 (left). The analysis reveals that the largest group comprises animals with a mixed lameness status.

### 3.2. Model Description

Our goal was to predict the lameness status based on the available data (characteristic variables). Specifically, we explored the feasibility of categorizing DIM, milk yield, and lactation stage into discrete classes to create robust models. Additionally, mean activity impulses per hour, mean lying (bout) duration, and averaged milk yield variables were computed, averaged over 3 days preceding the day of scoring. Finally, a season variable was introduced, taking into account possible seasonal dependence. The timing for seasons was defined as follows: Spring (1 March–31 May), Summer (1 June–31 August), Fall (1 September–30 November), and Winter (1 December–28/29 February). We considered a range of significance levels (from *p*-value <0.001 to 0.05). A detailed description is provided in Table 2.

In the following, we present a model that was developed and chosen from a range of alternative regression models (see Supplementary Materials) aiming to detect the lameness status based on the available data. We chose a logistic regression with mixed effects and different independent variables. The following independent variables were considered (see Table 1): mean activity impulses, mean lying bout duration, mean daily milk yield, DIM, parity, season, and their interactions.

We explored various options for random effects (refer to Supplementary Materials: models IV and V) and ultimately selected a random intercept dependent on individual cows. Additionally, interactions were taken into consideration in the model (see Table 2).

### 3.3. Model Predictions

To examine the ability of the implemented model to predict the lame/nonlame status, we divided the sample into training and validation datasets. We used the scoring by veterinarians available from our study according to [3] as a gold standard in the following calculations. To preserve the original characteristics of the data, the training dataset was extracted from the original data to include 80% of observations from each cow. Consequently, the training dataset comprises approximately 80% of the total observations. Once the model was trained on the training dataset, its predictive performance was assessed on the validation dataset, which contains the remaining 20% of the total observations. As mentioned earlier, the validation dataset consists of 20% of observations from each cow, making up approximately 20% of the original dataset. This approach was implemented to ensure that at least one observation from the same cow in the same farm is present in both the training and validation datasets, preserving the structure of the random effects component of the model.

The accuracy of prediction for the validation dataset, assessed as the number of correctly predicted values over the total number of predictions, was 0.81 (CI: 0.79–0.82). The precision (Pr=TP/(TP+FN)) of the model, indicating how well the model performs when a prediction is positive and, therefore, how many positive predictions are true, was 0.80. The recall (Rec=TP/(TP+FN+FP)) of the model, measuring how well the model predicts positive classes, was 0.78, calculated as the ratio of correct positive predictions to all positive classes. The weighted average for precision and recall, taking into account both false positives and false negatives, is the F1 (F1=Pr·Rec/(Pr+Rec)) score, which has a value of 0.82 for our predictions. Regarding sensitivity and specificity, characterizing the true-positive rate (similar to recall) and true-negative rate, and hence the proportion of truly classified positive and negative classes, the sensitivity and specificity for the validation dataset are 0.82 and 0.79, respectively.

For the training dataset, the AIC of the model is given as 13,625 and thus is much lower than the AIC of all other considered models (see Supplementary Materials).

**Figure 4:**
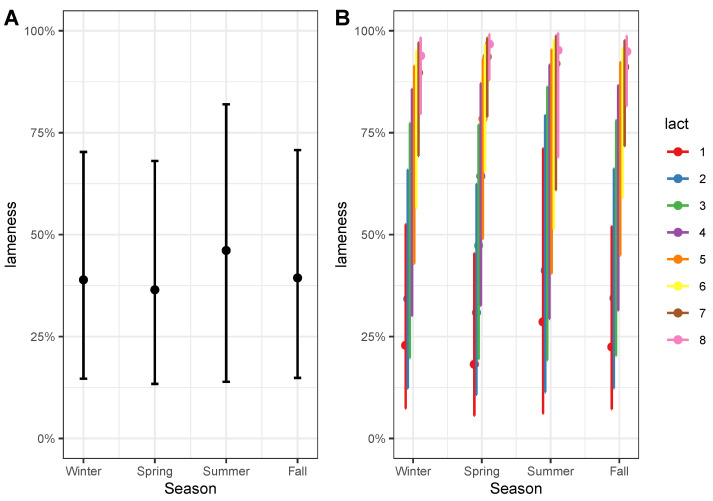
Probability of lameness in relation to the yearly seasons (**A**) and probability of lameness in relation to both the yearly seasons and the parity class (lact) (**B**).

With the help of the R package *ggeffects* [31], we were able to visualize the probability of lameness (Figure 4) predicted by the model. This visualization provides insights into the dependence of lameness probability on the independent variables and their interactions. These predictors were chosen on the basis of the calculated odds ratio values and *p*-values (see Table 2). For example, a high parity (variable “lact” in Table 1 and Table 2) class results in an almost 100% increase in lameness probability with an odds ratio (OR) of 1.890, CI (1.690,2.100), and a *p*-value of <10${}^{-16}$. On the other hand, the DIM variable appears not to significantly influence the curve slope of the dependence of lameness probability on the parity class, with an OR of 1.000, CI (0.996,1.003), and a slightly significant *p*-value of 0.089. However, the interaction term of both parity and DIM is slightly increased (OR=1.002, CI (0.998,1.004), and *p*-value = 0.066). It seems that parity is the most crucial factor for lameness, and this dependence may stem from considering parity as a proxy for age (see [32]). Regarding activity indicators, it appears that the activity impulses (per hour) averaged over 3 days preceding lameness scoring (OR=1.001, CI (0.995,1.002), *p*-value = 0.065) are slightly more important for lameness prediction than the average lying bout duration measured in minutes (OR=0.990, CI (0.990,1.002), *p*-value = 0.069). However, the interpretation of OR for continuous variables (activity impulses and lying duration) is dependent on units of measurement and requires caution. According to the calculated *p*-values in Table 2, the season factor is an important variable affecting lameness (especially in the interaction with lactation, e.g., for the interaction parity–Spring OR=1.140, CI (1.050,1.250) with a significant *p*-value of 0.003. However, as it can be seen from Figure 4, for the summer season, the spread in values is quite dramatic, and the probability to be lame varies approximately from 35 % to 70 % with large *p*-values (p> 0.1).

## 4. Discussion

We constructed a range of univariate models (see Supplementary Materials) for lameness detection using various variables (activity, milk yield, and cow-individual factors). Finally, we selected the model with the highest accuracy.

### 4.1. Interaction between Lameness Risk Factors and Seasons

The probability of lameness significantly decreases with the increase in daily milk yield, dropping from approximately 50% for low milk yield (5 kg) to 25 % for high milk yield (50 kg). This decrease might be explained by the correlation between lameness-causing illness and decreased milk yield. However, the seasonal interaction presents a different pattern, particularly in summer. In contrast with other seasons, lameness becomes more probable with increasing milk yield in the summer months. Cows in summer might suffer from heat stress, leading to delayed lameness following periods of heat stress [33]. Although the probability of lameness given by the model shows a broad variance, and the summer season factor is not statistically significant (Table 2) due to the limited number of observations in summer (Figure 3), it suggests a potential association. For other seasons, there is a slightly stronger decrease in lameness probability with higher milk yields in winter compared with spring and fall. Lower lameness probability is observed for increasing DIM. The increase in parity leads to curve saturation, with the probability of being lame approaching almost 100%. Similar dynamics are observed for the dependence of lameness probability on activity impulses and lying bout duration (not shown). The prediction of lameness becomes less reliable for extreme values of activity impulses (very low and very high) since the confidence interval for the “mean trend” widens. This may be due to various lameness risk factors, especially in summer, related to extreme temperatures [34]. However, in the present dataset, we have a small amount of data points (namely, 344) from summer, when most extreme temperature fluctuations occur. Therefore, the influence of summer could not be reliably assessed, as reflected in the high uncertainty in the odds ratio for this factor. This will be a subject of further investigations.

It is important to note that our analysis operates on the dataset as a whole, assuming stationarity on average. This approximation is motivated by data availability and the primary goal of the study: to reveal primary indicators of lameness, taking into account data from different cows and farms but without detailed time series analysis. Events well localized in time can affect instant lameness, but the longitudinal exploration of time series is beyond the scope of the present study. Future research should aim at analyzing the discovered indicators at a higher temporal resolution suitable for the analysis of nonstationary time series.

### 4.2. Quality of Fit of the Model

Our results demonstrate the potential of automated animal surveillance and promise to significantly improve lameness detection approaches in dairy livestock. With the daily activity data for cows from our study readily available, there is potential to extend the application of the developed model for lameness detection beyond the 3 or 7 days preceding scoring dates considered in this study.

In the implemented models, we neglected possible autocorrelations [33,34] or even cross-correlations of variables present on individual or farm levels. This is justified in Supplementary Materials, where we observed no significant improvement in the model’s quality when considering the activity on each of the few days preceding a lameness screening event instead of time-averaged activity indicators. Regarding the choice of variable types, such as nominal and ordinal, the analysis in Supplementary Materials suggests no substantial difference as well. It is worth mentioning that further behavioral characteristics, such as grooming [5] or even animal social behavior [35,36], might be utilized to assess lameness conditions. In this study, we divided cows into two groups—lame and nonlame—focusing on the most important risk factors for the detection and prediction of lameness. However, considering recent studies [37], it would be useful to perform statistical analysis and modeling by dividing animals into three groups: persistently nonlame, persistently lame, and those that may be lame or healthy interchangeably.

The future research goals will involve verifying the validity of the model, incorporating additional data, and extending the approach with promising machine learning techniques. These may include deep artificial neural networks [38,39,40], random forests, and boosted regression trees [41], especially as more and better time-resolved data become available.

In our study, the established scoring scheme after [3] was utilized as a gold standard for lameness assessment. While we acknowledge its limitations [8], an investigation of its validity is beyond the scope of the current study and should be analyzed separately.

## 5. Conclusions

Based on the analysis of widely available accelerometer activity data, alongside productivity indicators (milk yield) and cow-individual metadata, we constructed a statistical model (logistic regression with mixed effects) capable of detecting lameness in dairy cows with ≈80% sensitivity. The primary practical relevance, in our opinion, lies in the systematization of the main risk variables, encouraging farmers and veterinarians to closely monitor changes in these factors to prevent the development of lameness. Our findings also lay the groundwork for the design of computer-assisted decision support systems for automated surveillance and intervention management in the dairy industry.

## Figures and Tables

**Table 1:** Variables used in the statistical model (1).

Variable	Meaning
mean_impulses	Fixed effect referring to the individual cow’s activity (number of impulses) averaged over 3 days preceding the locomotion scoring
mean_lyi	Fixed effect referring to the individual cow’s activity (lying duration)averaged over 3 days preceding the locomotion scoring
mean_yield	Fixed effect referring to the individual cow’s developments of daily milk yield averaged over 3 days preceding the locomotion scoring
lact	Fixed effect of lactation number (parity) considered as a continuous variable
DIM	Fixed effects of the stage of lactation (days in milk) considered as a continuous variable
season	Fixed effect of season which can take values: Spring, Summer, Fall, and Winter
$cow_{i}$	Random effect (random intercept) of the *i*-th animal (i=[1…2757])
$farm_{j}$	Farm as random effect j=[1…6]
ε	Random residual

**Table 2:** Results for the logistic regression model with random effects (on the train dataset), using lameness status as the dependent variable. Predictor variables are explained in Table 1 corresponding to the model (1).

Fixed Effects	Estimate	Std. Error	Odds Ratios	OR CI	OR CI	*p*-Value	
				Lower	Upper		
Intercept	−1.144	7.223 $\times \;10^{-1}$	0.320	0.080	1.310	0.113	
mean_impulses	−2.011 $\times \;10^{-3}$	1.090 $\times \;10^{-3}$	1.001	0.995	1.002	0.065	.
mean_lyi	−5.070 $\times \;10^{-3}$	2.790 $\times \;10^{-3}$	0.990	0.990	1.002	0.069	.
lact	6.350 $\times \;10^{-1}$	5.570 $\times \;10^{-2}$	1.890	1.690	2.100	<2 $\times \;10^{-16}$	***
DIM	−1.480 $\times \;10^{-3}$	8.670 $\times \;10^{-4}$	1.000	0.996	1.003	0.089	.
mean_yield	1.150 $\times \;10^{-3}$	3.620 $\times \;10^{-3}$	1.001	0.990	1.010	0.751	
season_Spring	−6.330 $\times \;10^{-1}$	3.070 $\times \;10^{-1}$	0.530	0.290	0.970	0.039	*
season_Summer	−9.560 $\times \;10^{-1}$	1.680	0.380	0.010	1.029	0.569	
season_Fall	−1.270	0.350	3.930 $\times \;10^{-1}$	0.130	0.610	0.001	**
mean_impulses:season_Spring	2.640 $\times \;10^{-4}$	1.570 $\times \;10^{-3}$	1.001	0.997	1.003	0.867	
mean_impulses:season_Summer	−2.670 $\times \;10^{-3}$	4.940 $\times \;10^{-3}$	0.993	0.984	1.003	0.589	.
mean_impulses:season_Fall	5.210 $\times \;10^{-3}$	1.830 $\times \;10^{-3}$	1.002	0.999	1.001	0.004	**
mean_lyi:DIM	1.840 $\times \;10^{-5}$	8.958 $\times \;10^{-6}$	1.001	1.002	1.010	0.070	.
mean_lyi:season_Spring	1.700 $\times \;10^{-3}$	2.590 $\times \;10^{-3}$	1.002	1.002	1.010	0.513	
mean_lyi:season_Summer	7.440 $\times \;10^{-3}$	1.360 $\times \;10^{-2}$	1.010	0.980	1.030	0.584	
mean_lyi:season_Fall	6.040 $\times \;10^{-3}$	3.180 $\times \;10^{-3}$	1.010	1.002	1.010	0.057	.
lact:DIM	−8.330 $\times \;10^{-4}$	2.110 $\times \;10^{-4}$	1.002	0.998	1.004	0.066	.
lact:season_Spring	1.340 $\times \;10^{-1}$	4.520 $\times \;10^{-2}$	1.140	1.050	1.250	0.003	**
lact:season_Summer	−5.760 $\times \;10^{-2}$	1.700 $\times \;10^{-1}$	0.940	0.680	1.320	0.735	
lact:season_Fall	−1.080 $\times \;10^{-2}$	5.300 $\times \;10^{-2}$	0.990	0.890	1.100	0.839	
mean_yield:season_Spring	2.720 $\times \;10^{-3}$	4.790 $\times \;10^{-3}$	1.002	0.990	1.010	0.569	
mean_yield:season_Summer	4.420 $\times \;10^{-2}$	2.760 $\times \;10^{-2}$	1.050	0.990	1.100	0.109	
mean_yield:season_Fall	7.630 $\times \;10^{-3}$	6.690 $\times \;10^{-3}$	1.010	0.999	1.020	0.254	
**Random effects**							
	**Groups**		**Names**		**Variance**		**Std. Error**
	cow:farm		Intercept		2.32		1.52
	farm		Intercept		2.58		1.61

Significance codes: 0 ‘***’ 0.001 ‘**’ 0.01 ‘*’ 0.05 ‘.’ 0.1 ‘ ’ 1.

## Data Availability

The raw data can be obtained upon a reasonable request from the consortium participating in KlauenFitnet 1.0 and 2.0.

## Supplementary Materials

The following supporting information can be downloaded at https://www.mdpi.com/article/10.3390/ani13233681/s1: Table S1. Calculated coefficients for linear regression model; Table S2. Calculated coefficients for logistic regression model I; Table S3. Calculated coefficients for logistic regression model III; Table S4. Calculated coefficients for logistic regression model with mixed effects; Table S5. Calculated coefficients for logistic regression model V.
