# Supplementary material for: Leveraging Accelerometer Data for Lameness Detection in Dairy Cows: A Longitudinal Study of Six Farms in Germany
Source: Animals (Basel). 2023 Nov 28;13(23):3681. doi: 10.3390/ani13233681 (PMC10705170; doi:10.3390/ani13233681)
Supplement: Supplementary file 1 [file animals-13-03681-s001.zip › animals-2629024-supplementary.pdf]

Supplementary Material

1 Supplementary Data

The parity class and the days in milk (DIM) variables were transformed into categorical variables, with categories 1, 2, 3, 4+ (lact_cat) and <50, 51-100, 101-200, 201-305, 305+ (DIM_cat) respectively. The milk yield values were aggregated creating first a numerical milk yield variable containing the mean of milk yield for the three days preceding locomotion scoring and then grouping them into three categories (MKL): low milk yield (mean< 28.8 kg), medium (mean between 28.9 kg and 38.4 kg) and high (mean greater than 39 kg).

2 Linear regression model

The variables lact_cat, DIM_cat and MKL were taken as ordered categorical variables. Also the model incorporates all the lagged variables for steps number, lying bout duration for 3 days before scoring as well as seasonal factors. The lameness variable y_{it}- individual value of the locomotion score is considered a continuous numerical variable and modelled according to the equation:

$$y_{it} = x_{it}\beta + \varepsilon, \tag{1}$$

with β being a vector of regression coefficients (fixed effects) and x_{it} being a matrix of major independent or explanatory variables:

- **day_act**$_j$ -fixed effect referring to the individual cow's activity (number of steps) accounting for the j-th day until locomotion scoring (j=[1...3]),
- **day_lyi**$_j$ - fixed effect referring to the individual cow's activity (lying duration) accounting for the j-th day until locomotion scoring (j=[1...3])
- **day_milk**$_j$ fixed effect referring to the individual cow's daily milk yield accounting for the j -th day until locomotion scoring (j=[1...3])
- **lact**$_k$ fixed effects of the k-th parity class (k=[1...3], parities 1, 2, 3 and >3),
- **DIM**$_l$ fixed effects of the stage of lactation or days in milk (if used as categorical variables l=[1...5] ; locomotion scoring on days 1- 50, 51-100, 101-200, 201-305,>305 after calving),
- ε - random residual

The results of the univariate linear regression (S1) without fixed effects (performed with **lm** function in R) are presented in Table S1). Coefficient of determination as as measure of correlation between theoretically predicted and real lameness score values R^2 was equal to 0.12, implying a weak prediction strength of the model. The calculated AIC equals to 56131. It should be noted that increasing the number of days (7 and more) before scoring for averaging didn't lead to significant R^2 change, so in further models we have considered 3 days before scoring.

2.1 Logistic regression models without taking into account of seasonality

2.1.1 Logistic regression model I

For this model we have used variables discussed in the main text. In the first version of the model we have considered milk yield and lactation periods as categorical variables. Obtained values (we have used function **glm()** in R) for coefficients and statistical parameters are presented in Table S2.

2.1.2 Logistic regression model II

We also performed logistic regression considering DIM and parity as categorical variables. It should be noted that the aggregation of DIM and parity lead to a lower AIC (24437) in comparison with the model where these variables were continuous (the AIC=24501).

2.2 Logistic regression models with seasonality

2.2.1 Logistic regression model III

Based on the exploratory data (Fig. 3, main text) and knowing that the impact of high temperature in summer months could lead up to 20% changes in the activity of dairy cows in Germany, Heinicke et al (2019), Heinicke et al (2018), we included a seasonal variable into the logistic model, see Table S3.

From the model, all the predictors seem to be significant with the only exception of the DIM variable (days in milk class), or at least parts of its categories. To notice that the mean number of steps and the mean lying frequency have a negative impact on the lameness, i.e. for every change unit in mean steps the log odds of being lame (vs not being lame) decreases by 0.005. For the categorical variables, for example LKL (lactation number): being in a second lactation vs being in a first, changes the log odds of being lame by 0.623, being in a third vs being in a first by 1.11 and so on. Anyway, as expected, this model performs poorly in terms of goodness of fit, where is calculated R^2 by different methods gives following values: 0.08(Hosmer and Lemeshow), 0.105 (Cox and Snell), 0.14(Nagelkerke).

One way to improve this model is to handle the structure of the data properly. Hence, a logistic regression with mixed effect model was further used.

2.3 Logistic regression with mixed effects

2.3.1 Logistic regression model IV

The model includes all the three lags for the number of steps as well as for the lying bout duration together with the categorical variables for the lactation, days in milk, milk yield and season, see Table S4. For this model we have considered the variable milk yield as categorical (see also the description at the beginning of Supplementary). Compared to the first model, the AIC is lower then in the previous models and the goodness of fit for the model (conditional R^2=0.66, marginal R^2=0.074) increased as expected. Thus incorporation of random effects to the model increased the goodness of fit up to 66%.

2.3.2 Logistic regression model V

The milk yield for this model was treated as a numerical variable instead of categorical one, showing an increase in the significance for this variable, see Table S5. Overall the model performs with R^2=67.5% that is slightly increased value in comparison with R^2 for previous one (model IV).

Table S1: Calculated coefficients for linear regression model (S1). The activity of cows in the model was taken into account as the number of steps (day_act) and the lying bout duration (day_lyi) measured for one, two and three days before locomotion scoring. The milk yield value (day_milk) was also included in the model. The variables DIM and cow parity class (lact_cat) were considered as categorical. The variable DIM_cat corresponds to number of days since calving at the time of the locomotion scoring and intervals 0–50, 51–100, 101–200, 201–305 and more than 305 were chosen for each level. Cow parity is represented by four levels correspond to lact_cat1, lact_cat2, lact_cat3, lact_cat4 (for all parities > than 3).

**Coefficients**	**Estimate**	**Std. Error**	**p–value**	
Intercept	3.700	0.042	2e-16	***
day_act3	-1e-03	1e-04	2.3e-14	***
day_act2	-7e-04	2e-04	5.1e-06	***
day_act1	-1e-03	1e-04	2e-16	***
DIM_cat>305	-0.083	0.030	0.005	**
DIM_cat 101-200	0.036	0.0270	0.184	
DIM_cat 201-305	-0.107	0.028	1e-04	***
DIM_cat 51-100	0.178	0.0296	2.1e-09	***
lact_cat2	-0.703	0.0205	2e-16	***
lact_cat3	-0.351	0.021	2e-16	***
lact_cat4	-0.167	0.021	4.9e-15	***
day_milk1	-0.009	0.001	3.0e-10	***
day_milk2	-0.004	0.001	0.002	**
day_milk3	-0.003	0.001	0.045	*
day_lyi1	-1e-04	3e-04	0.629	
day_lyi2	1e-04	3e-04	0.670	
day_lyi3	-8e-04	3e-04	0.008	***

Significance codes: 0 '***' 0.001 '**' 0.01 '*' 0.05 '.' 0.1 ' ' 1

Table S2: Calculated coefficients for logistic regression model I (all factors are the same as for table S1)

**Coefficients**	**Estimate**	**Std. Error**	***p*–value**	
Intercept	3.700	0.096	2e-16	***
day_act3	-0.001	0.0001	2e-16	***
day_act2	-0.0007	0.0002	5.1e-06	***
day_act1	-0.001	0.0003	1.7e-06	***
DIM_cat>305	0.178	0.0296	2.1e-09	***
DIM_cat101-200	0.04	0.03	0.017	
DIM_cat201-305	-0.107	0.0279	0.00013	***
DIM_cat51-100	-0.083	0.030	0.005	**
lact_cat2	-0.703	0.021	2e-16	***
lact_cat3	-0.351	0.021	2e-16	***
lact_cat4	-0.167	0.021	4.9e-15	***
day_milk1	-0.0087	0.0014	3.0e-10	***
day_milk2	-0.0038	0.0013	0.002	**
day_milk3	-0.003	0.001	0.05	*
day_lyi1	-0.0001	0.0003	0.63	
day_lyi2	0.0001	0.0003	0.67	
day_lyi3	-0.0008	0.0007	0.008	**

Significance codes: 0 '***' 0.001 '**' 0.01 '*' 0.05 '.' 0.1 ' ' 1

Table S3: Calculated coefficients for logistic regression model III.The activity of cows in the model was taken into account as the mean number of steps (mean_act) and the mean lying bout duration (mean_lyi) calculated for three days before locomotion scoring. The milk yield value (day_milk) was also included in the model averaged for three days before scoring. The variables DIM and cow parity class (lact_cat) were considered as categorical. The variable DIM_cat corresponds to number of days since calving at the time of the locomotion scoring and intervals 0–50, 51–100, 101–200, 201–305 and more than 305 were chosen for each level. Cow parity is represented by four levels correspond to lact_cat1, lact_cat2, lact_cat3, lact_cat4 (for all parities > than 3). Seasonal variables were taken into account as Spring, Fall and Summer.

**Coefficients**	**Estimate**	**Std. Error**	***p*–value**	
Intercept	1.12	0.1	<2e-16	***
mean_steps	-0.007	0.0004	<2e-16	***
mean_lyi	-0.006	0.0007	<2e-16	***
lact_cat2	0.61	0.041	<2e-16	***
lact_ cat3	1.108	0.043	<2e-16	***
lact_cat4	1.367	0.047	4.9e-15	***
DIM_cat51-100	0.4128	0.069	2.2e-09	***
DIM_cat101-200	0.17	0.06	0.017	**
DIM_cat201-305	-0.096	0.06	0.0062	
DIM_cat>305	-0.04	0.07	0.59	
milk_yield	-0.026	0.001	<2e-16	***
Season_Spring	0.11	0.04	0.001	**
Season_Summer	-0.995	0.142	2.7e-12	***
Season_Fall	0.384	0.042	<2e-16	***

Significance codes: 0 '***' 0.001 '**' 0.01 '*' 0.05 '.' 0.1 ' ' 1

AIC: 25890

Table S4: Calculated coefficients for logistic regression model with mixed effects (S4). The activity of cows in the model was taken into account as the number of steps (day_act) and the lying bout duration (day_lay) measured for one, two and three days before locomotion scoring. The milk yield value (day_milk) was also included in the model. The variables DIM cow parity class (lact_cat) and milk yield (MKLM and MKHL) were considered as categorical. The variable DIM_cat corresponds to number of days since calving at the time of the locomotion scoring and intervals 0–50, 51–100, 101–200, 201–305 and more than 305 were chosen for each level. Cow parity is represented by four levels correspond to lact_cat1, lact_cat2, lact_cat3, lact_cat4 (for all parities > than 3). MKLM and MKHL mean <30 kg and >30 kg milk yield per day correspondingly. Seasonal variables were taken into account as Spring, Fall and Summer.

**Coefficients**	**Estimate**	**Std. Error**	***p*–value**	
Intercept	-0.619	0.182	0.0007	***
day_act1	-0.001	0.0006	0.03	*
day_act2	0.00015	0.0007	0.83	
day_act3	-0.002	0.0006	0.007	**
day_lyi1	-4.4e-04	1e-03	0.68	
day_lyi2	-0.002	0.001	0.08	.
day_lyi3	-6.2e-04	0.001	0.56	
lact_cat2	0.86	0.12	<2e-12	***
lact_cat3	1.58	1.13	<2e-16	***
lact_cat4	2.04	0.15	<2e-16	***
DIM_cat51-100	0.28	0.097	0.004	**
DIM_cat101-200	0.09	0.096	0.35	
DIM_cat201-305	-0.096	0.06	0.03	*
DIM_cat>305	-0.28	0.12	0.02	*
MKLM	0.03	0.06	0.68	
MKLH	-0.01	0.08	0.89	
Season_Spring	0.04	0.05	0.49	
Season_Summer	0.19	0.25	0.47	
Season_Fall	0.05	0.06	0.38	

Random effects

**Groups**	**Names**	**Variance**	**Std.Error**
cow	Intercept	5.68	2.38

Significance codes: 0 ’***’ 0.001 ’**’ 0.01 ’*’ 0.05 ’.’ 0.1 ’ ’ 1

AIC: 19075

Table S5: Calculated coefficients for logistic regression model V. Variables were used the same as in model III.

**Coefficients**	**Estimate**	**Std. Error**	***p*–value**	
Intercept	-0.11	0.19	0.55	
mean_steps	-0.003	0.0008	0.0002	***
mean_lyi	-0.003	0.001	0.03	*
lact_cat2	0.907	0.120	3.4e-14	***
lact_cat3	1.650	1.132	<2e-16	***
lact_cat4	2.107	0.148	<2e-16	***
DIM_cat51-100	0.278	0.097	0.004	**
DIM_cat101-200	0.048	0.095	0.004	
DIM_cat201-305	-0.096	0.06	0.03	***
DIM_cat>305	-0.477	0.122	1e-04	***
milk_yeld	-0.017	0.003	6.3e-10	***
Season_Spring	-0.03	0.05	0.5	
Season_Summer	0.20	0.25	0.42	
Season_Fall	0.06	0.06	0.30	.

**Random effects**	**Variance**	**Std. Error**	
**Groups**	**Names**	**Variance**	**Std.Error**
cow	Intercept	5.42	2.33

Significance codes: 0 '***' 0.001 '**' 0.01 '*' 0.05 '.' 0.1 ' ' 1

AIC: 19016
